# ActDES – a curated Actinobacterial Database for Evolutionary Studies

**DOI:** 10.1099/mgen.0.000498

**Published:** 2021-01-12

**Authors:** Jana K. Schniete, Nelly Selem-Mojica, Anna S. Birke, Pablo Cruz-Morales, Iain S. Hunter, Francisco Barona-Gomez, Paul A. Hoskisson

**Affiliations:** ^1^​ Biology Department, Edge Hill University, St Helens Road, Ormskirk, Lancashire L39 4QP, UK; ^2^​ Strathclyde Institute of Pharmacy and Biomedical Sciences, University of Strathclyde, 161 Cathedral Street, Glasgow G4 0RE, UK; ^3^​ Evolution of Metabolic Diversity Laboratory, Langebio, Cinvestav-IPN, Libramiento Norte Carretera Leon Km 9.6, 36821 Irapuato, Guanajuato, México

**Keywords:** biosynthetic gene cluster, evolution, natural product, primary metabolism, specialized metabolism, *Streptomyces*

## Abstract

*
Actinobacteria* is a large and diverse phylum of bacteria that contains medically and ecologically relevant organisms. Many members are valuable sources of bioactive natural products and chemical precursors that are exploited in the clinic and made using the enzyme pathways encoded in their complex genomes. Whilst the number of sequenced genomes has increased rapidly in the last 20 years, the large size, complexity and high G+C content of many actinobacterial genomes means that the sequences remain incomplete and consist of large numbers of contigs with poor annotation, which hinders large-scale comparative genomic and evolutionary studies. To enable greater understanding and exploitation of actinobacterial genomes, specialized genomic databases must be linked to high-quality genome sequences. Here, we provide a curated database of 612 high-quality actinobacterial genomes from 80 genera, chosen to represent a broad phylogenetic group with equivalent genome re-annotation. Utilizing this database will provide researchers with a framework for evolutionary and metabolic studies, to enable a foundation for genome and metabolic engineering, to facilitate discovery of novel bioactive therapeutics and studies on gene family evolution. This article contains data hosted by Microreact.

## Data Summary

1. All genome sequences used in this study can be found in the National Center for Biotechnology Information (NCBI) Taxonomy Browser (https://www.ncbi.nlm.nih.gov/Taxonomy/Browser/wwwtax.cgi) and are summarized along with accession numbers in Table S1 (available on Figshare – https://doi.org/10.6084/m9.figshare.13143407.v1). Other data are available on Figshare (https://doi.org/10.6084/m9.figshare.13143407.v1).

2. Perl script files are available on GitHub (https://github.com/nselem/ActDES), including details of how to batch annotate genomes in rast from the terminal (https://github.com/nselem/myrast).

3. Table S1 shows a list of genomes from the NCBI (actinobacteria database.xlsx) and is available on Figshare (https://doi.org/10.6084/m9.figshare.13143407.v1).

4. CVS genome annotation files including the fasta files of nucleotide and amino acids sequences (individual .cvs files) are available on Figshare (https://doi.org/10.6084/m9.figshare.13143407.v1).

5. blast nucleotide database (.fasta file) information is available on Figshare (https://doi.org/10.6084/m9.figshare.13143407.v1).

6. blast protein database (.fasta file) information is available on Figshare (https://doi.org/10.6084/m9.figshare.13143407.v1).

7. Table S2 expansion table – genus level (expansion table.xlsx – tab genus level) is available on Figshare (https://doi.org/10.6084/m9.figshare.13143407.v1).

8. Table S2 expansion table – species level (expansion table.xlsx – tab species level) is available on Figshare (https://doi.org/10.6084/m9.figshare.13143407.v1).

9. All GlcP and Glk data – blast hits from ActDES, muscle alignment files and .nwk tree files – can be found on Figshare (https://doi.org/10.6084/m9.figshare.13143407.v1).

10. Interactive trees in Microreact for Glk (https://microreact.org/project/w_KDfn1xA/5a178533) and associated files can be found on Figshare (https://doi.org/10.6084/m9.figshare.13143407.v1).

11. Interactive trees in Microreact for GlcP (https://microreact.org/project/VBUdiQ5_k/045c95e1) and associated files can be found on Figshare (https://doi.org/10.6084/m9.figshare.13143407.v1).

12. Jupyter Notebook for exploring ActDES in MyBinder can be found at https://github.com/nselem/ActDES.

**Significance as a Bioresource to the Community**

The *Actinobacteria* is a large diverse phylum of bacteria, often with large, complex genomes with a high G+C content. Sequence databases have great variation in the quality of sequences, equivalence of annotation and phylogenetic representation, which makes it challenging to undertake evolutionary and phylogenetic studies. To address this, we have assembled a curated, taxa-specific, non-redundant database to aid detailed comparative analysis of *Actinobacteria *. ActDES (Actinobacterial Database for Evolutionary Studies) constitutes a novel resource for the community of actinobacterial researchers that will be useful primarily for two types of analyses: (i) comparative genomic studies, facilitated by reliable identification of orthologs across a set of defined phylogenetically representative genomes, and (ii) phylogenomic studies, which will be improved by identification of gene subsets at specified taxonomic level. These analyses can then act as a springboard for the studies of the evolution of virulence genes, the evolution of metabolism and identification of targets for metabolic engineering.

## Introduction

The increase in availability of bacterial whole-genome sequencing provides large amounts of data for evolutionary and phylogenetic analysis. However, there is great variation in the quality, annotation and phylogenetic skew of the data available in large universal databases, meaning that evolutionary and phylogenetic studies can be challenging. To address this variation, curated, high-level, taxa-specific, non-redundant sub-databases need to be assembled to aid detailed analysis. Given that there is a direct correlation between phylogenetic distance and the discovery of novel function [1–3], it is imperative that any derived databases must be phylogenetically representative and non-redundant to enable insight into the evolution of genes, proteins and pathways within a given group of taxa [1].

The phylum *Actinobacteria* is a major taxon amongst the *Bacteria*, which includes phenotypically and morphologically diverse organisms found on every continent and in virtually every ecological niche [4]. They are particularly common in soils, yet within their ranks are potential human and animal pathogens such as *Corynebacterium *, *Mycobacterium *, *Nocardia* and *Tropheryma *, inhabitants of the gastrointestinal tract (* Bifidobacterium* and *Scardovia *), as well as plant commensals and pathogens such as *Frankia *, *Leifsonia* and *Clavibacter* [4, 5]. Perhaps the most notable trait of the phylum is the renowned ability to produce bioactive natural products such as antibiotics, anti-cancer agents and immuno-suppressive agents, with genera such as *Amycolatopsis *, *Micromonospora* and *Streptomyces* being particularly prominent [6]. As a result, computational ‘mining’ of actinobacterial genomes has become an important part of the drug-discovery pipeline, with increasing numbers of online resources and software devoted to identification of natural-product biosynthetic gene clusters (BGCs) [7–9]. It is important to move beyond approaches that rely on similarity searches of known BGCs and to expand searches to identify hidden chemical diversity within the genomes [6, 7, 10–13].

A recent study of 830 actinobacterial genomes found >11 000 BGCs comprising 4122 chemical families, indicating that there is a vast diversity of strains and chemistry to exploit [14], yet within each of these strains there will be hidden diversity in the form of cryptic BGCs. To exploit this undiscovered diversity as the technology develops and databases expand, new biosynthetic logic will emerge, yet we know little of how natural selection shapes the evolution of BGCs and how biosynthetic precursors are supplied to gene products of BGCs from primary metabolism and to identify targets for metabolic engineering of industrially relevant strains. Such logic will expedite industrial strain improvement processes, enabling titre increases and development of novel molecules, as well as the engineering of strains to use more sustainable feedstocks.

To aid this process, we have created an actinobacterial metabolism database including functional annotations for enzymes from 612 species to enable phylum-wide interrogation of gene expansion events that may indicate adaptive evolution, help shape metabolic robustness for antibiotic production [15] or enable the identification of targets for metabolic engineering. Actinobacterial Database for Evolutionary Studies (ActDES) provides a curated list of high-quality, phylum-specific genomes and data to help users navigate the redundancy and inconsistency in sequence databases in a simplified format that enables researchers with little taxonomic knowledge to develop testable evolutionary hypotheses. To demonstrate the utility of ActDES, we have detailed its construction and used it to investigate the glucose permease/glucokinase system phylogeny across the *Actinobacteria *.

## Methods

**Fig. 1.**
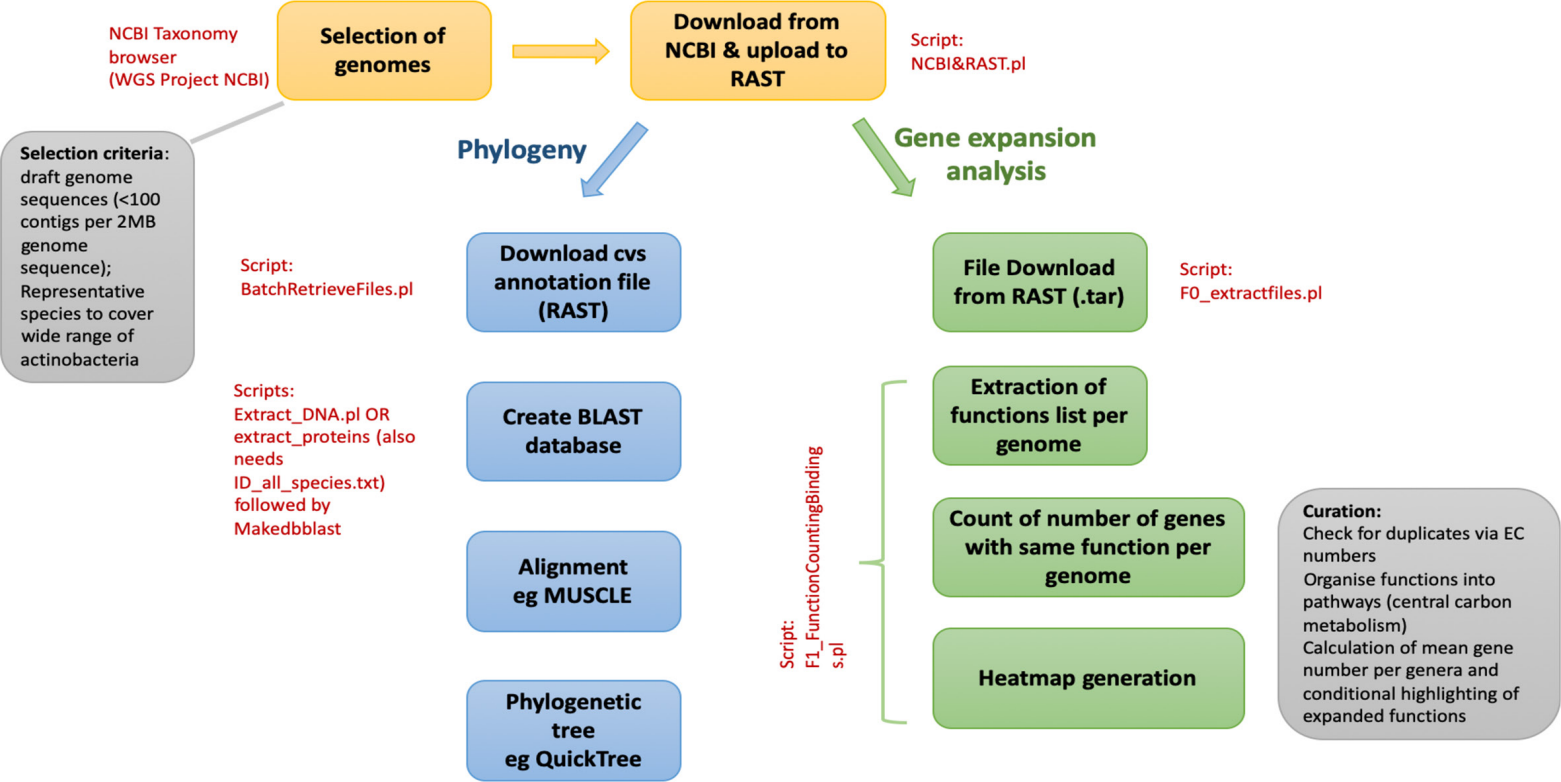
Schematic workflow for the creation of ActDES. Genomes were selected from NCBI Taxonomy Browser and uploaded for annotation to rast [38]. The annotated genomes were then processed for two different analyses. Firstly, the functional roles were downloaded and for each functional role the numbers of occurrences per genome were counted in order to obtain an expansion table (Table S2) by comparing the mean of each genus to the overall mean of all genera. Secondly, the genomes were used to extract all nucleotide and protein sequences in fasta format, which could then be queried by sequence using blast [39]. The hits were aligned in muscle [16] and after refinement the alignment was used to reconstruct phylogenetic trees in QuickTree [17].

We generated ActDES, a database for evolutionary analysis of actinobacterial genomes, in two formats: a database for interrogation by blastn or blastp for phylogenetic analysis, and a primary metabolic gene expansion table, which can be mined at different taxonomic levels (Tables S1 and S2) for specific metabolic functions from primary metabolism. A schematic overview of the generation of the dataset is shown in Fig. 1.

The database was generated via the National Center for Biotechnology Information (NCBI) Taxonomy Browser (https://www.ncbi.nlm.nih.gov/Taxonomy/Browser/wwwtax.cgi) to identify actinobacterial genome sequences. The quality of the genome sequences was filtered by the number of contigs (<100 contigs per 2 Mb of genome sequence) and the genomes were downloaded from the NCBI WGS repository (https://www.ncbi.nlm.nih.gov/Traces/wgs/). These genomes were then dereplicated to ensure that the database comprised a wide taxonomic range of the phylum, resulting in 612 species from 80 genera within 13 suborders of the *Actinobacteria* (Table S1).

Each of these 612 genomes was reannotated using rast. Default settings were used to ensure equivalence of annotation across the database and the annotation files of each genome were downloaded (cvs files – https://doi.org/10.6084/m9.figshare.13143407.v1). These annotation files were subsequently used to extract all protein and nucleotide sequences into two files. Each of these files was subsequently converted into blast databases (a protein database and a nucleotide database – https://doi.org/10.6084/m9.figshare.12167724) to facilitate phylogenetic analysis. Sequences of interest can be aligned using muscle [16] and phylogenetic trees reconstructed using a range of tree construction software such as QuickTree [17], IQ tree [18] or MrBayes [19]. Subsequent trees may be visualized in software such as FigTree v1.4.2 (http://tree.bio.ed.ac.uk/software/ﬁgtree/).

The rast annotation files were also used to extract the functional roles of each coding sequence (CDS) per genome and the level of gene expansion was assessed for each genome by counting the number of genes per species per functional category (gene function annotation). The dataset was then curated manually for central carbon metabolism and amino acid biosynthesis pathways to create the gene expansion table (Table S2), with the organisms grouped according to their taxonomic position. The quality of the data was checked at each step for duplicates and inconsistencies, and was curated manually to exclude faulty entries. As the NCBI Taxonomy Browser database is overrepresented in *Streptomyces* genomes due to the number of species that have been sequenced relative to other *Actinobacteria *, this is also reflected in ActDES (288 *Streptomyces* genomes from a total of 612 genomes). However, this was addressed in the expansion table (Table S2) by calculating the mean occurrence of each functional category within each genus and then calculating an overall mean for the phylum to compensate. The mean occurrence of each functional category per genus plus the standard deviation was also calculated, and this was used to analyse the occurrence of each functional gene category per species within Table S2. A gene function annotation with a gene copy number value above the mean plus the standard deviation for each genus indicated that there had been a gene expansion event in that species and this was noted. The gene expansion table (Table S2) enables researchers to identify groups of genes of interest for subsequent phylogenetic and evolutionary analysis, which can be performed with confidence due to the highly curated nature of the data included in the database.

As the NCBI Taxonomy Browser database is overrepresented in *Streptomyces* genomes due to the number of species that have been sequenced relative to other *Actinobacteria *, this is also reflected in ActDES (288 *Streptomyces* genomes from a total of 612 genomes). However, this was addressed in the expansion table (Table S2) by calculating the mean occurrence of each functional category within each genus and then calculating an overall mean for the phylum to compensate. The mean occurrence of each functional category per genus plus the standard deviation was also calculated, and this was used to analyse the occurrence of each functional gene category per species within Table S2. A gene function annotation with a gene copy number value above the mean plus the standard deviation for each genus indicated that there had been a gene expansion event in that species and this was noted. The gene expansion table (Table S2) enables researchers to identify groups of genes of interest for subsequent phylogenetic and evolutionary analysis, which can be performed with confidence due to the highly curated nature of the data included in the database.

## Results

The gene expansion table (Table S2) lists 612 species of 80 genera within the *Actinobacteria* with data that provides an extensive analysis at the phylum level, which is the starting point for detailed phylogenomic studies. Gene expansions were identified in separate datasets at the genus and species levels, along with details of the numbers of genes in each functional category per species and the mean numbers of genes in each functional category per genus expanded within the genomes. These data can be used subsequently in phylogenomic analyses to identify targets for metabolic engineering and gene function studies. Identification of expanded gene families may also facilitate the recognition of novel natural product BGCs, for which gene expansion events of primary metabolic genes have been classified to be associated within BGCs as biosynthetic enzymes or through provision of additional copies of antibiotic targets that may subsequently function as resistance mechanisms [6, 11, 20–24].

This database has found utility for studying primary metabolic gene expansions in *Streptomyces *. It enabled a detailed *in silico* analysis of the duplication event leading to the two pyruvate kinases in the genus of *Streptomyces *, subsequently enabling the functional characterization of the two isoenzymes to reveal how they contribute to metabolic robustness [15]. ActDES may also be useful for investigating the distribution of primary metabolic genes across the phylum to link phenotype to genotype and phylogenetic position. An initial RpoB phylogeny has been reconstructed previously using this database [15], which provided a robust universal phylogeny for comparison of individual protein trees [25].

**Fig. 2.**
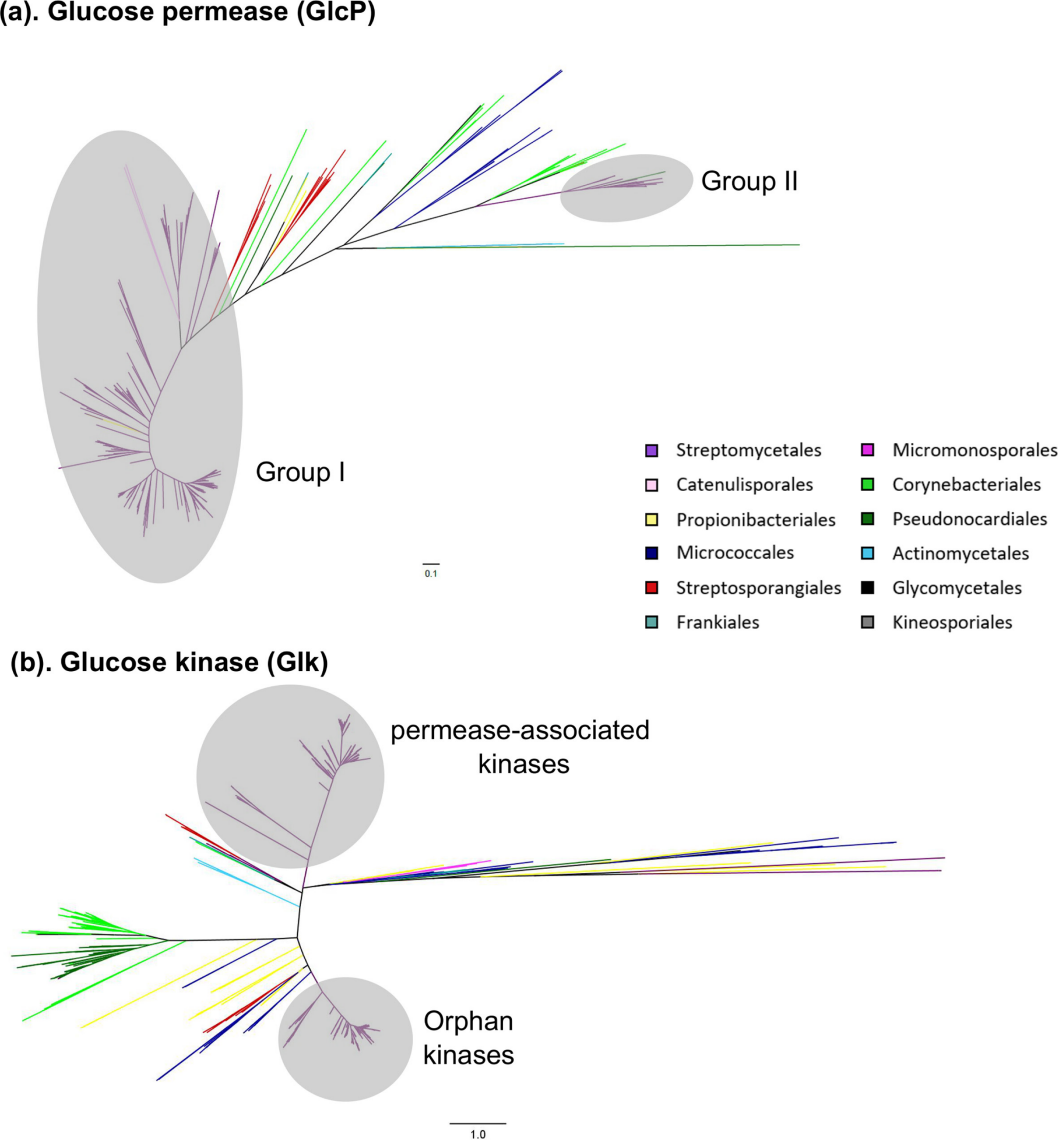
(a) Actinobacterial-wide phylogenetic tree of glucose permeases (GlcP) and (b) actinobacterial-wide phylogenetic tree of glucokinases (Glk). Trees are colour-coded according to the NCBI Taxonomy Browser (https://www.ncbi.nlm.nih.gov/Taxonomy/Browser/wwwtax.cgi). Interactive trees are also available via Microreact [29]: GlcP –https://microreact.org/project/VBUdiQ5_k/045c95e1, and Glk – https://microreact.org/project/w_KDfn1xA/5a178533. Scale bar indicates branch lengths equivalent to one substitution per site.

To demonstrate the utility of ActDES, the glucose permease/glucokinase system of the *Actinobacteria* was investigated. The role of nutrient-sensing in regulation of antibiotic biosynthesis is well known [26], with the enzyme glucokinase (Glk) playing a central role in carbon-catabolite repression (CCR) in *Streptomyces* [27]. In most bacteria, CCR is mediated by the phosphoenolpyruvate-dependent phosphotransferase system (PTS), yet in *Streptomyces,* glucose uptake is mediated by the major-facilitator superfamily (MFS) transporter, glucose permease (GlcP), and there is evidence for direct interaction between Glk and GlcP, which may mediate CCR [28]. Understanding the nature and distribution of these enzymes will play a key role in developing industrial fermentations with glucose as major carbon source. Investigating the distribution of the glucose permease/glucokinase system across the phylum shows that GlcP and Glk have been the subject of gene expansion events in some members of the *Streptomycetales *, most notably the *Streptomyces *, with a patchy distribution of the Glk/GlcP system across the remainder of the phylum (Table S2; genus tab). However, where the Glk/GlcP system is found, the number of expansion events observed is greater for Glk than for GlcP (Fig. 2a, b). The phylogenetic trees (Fig. 2a, b) clearly show two clades for Glk and GlcP within the *Streptomycetales* (interactive trees are available via Microreact [29]: Glk – https://microreact.org/project/w_KDfn1xA/5a178533, and GlcP – https://microreact.org/project/VBUdiQ5_k/045c95e1). However, these clades differ in the number of sequences, with the Glk clades being equal in number, suggesting that a duplication event has occurred within the *Streptomycetales* (Fig. 2b). The is consistent throughout the order, with the patterns largely the same as observed for *Streptomyces coelicolor *. This species has two ROK-family ATP-dependent glucokinases, SCO2126 (*glkA*) and SCO6260, that share around 50 % amino acid sequence identity, and each is found in one of the distinct clades (permease-associated kinases and orphan kinases (Fig. 2b). Whilst SCO2126 is a GlcP-associated kinase, the gene encoding SCO6260 is located in an operon including genes encoding a putative carbohydrate ABC-transporter system, which has been reported elsewhere [30]. SCO6260 appears to be the only glucokinase in the database that is associated with an ABC-transporter. This may suggest that expansion of the Glk gene family in *Streptomycetales* might have occurred to extend the number of CCR-mediating kinases in the genome, adding increased regulatory complexity to carbohydrate metabolism in this group of organisms that use CCR as a major regulator of specialized metabolism.

The two clades for GlcP within the *Streptomycetales* differ in size, suggesting either gene duplication followed by gene loss or an expansion through horizontal gene transfer (HGT) has occurred. A detailed examination of these clades by species (Table S2; species tab) shows the presence of both scenarios. There are duplicated enzymes located within the same clade (as observed in *S. coelicolor *; group I) or additional copies of the permease that are located in a phylogenetically distinct clade, which lacks congruence with the RpoB tree [15], and remarkably consists entirely of sequences from the genus *Streptomyces* (group II; Fig. 2a). This suggests that they may have been acquired via HGT. The expansive nature of the duplicated Glk enzymes compared to GlcP may be due to the role played in CCR by the GlkA enzymes [27], and the different transcriptional activities under glycolytic and gluconeogenic conditions [31], yet quite how these different Glk enzymes interact with the permease(s) under various conditions requires further experimental investigation to understand their exact physiological role, and how this may be translated into industrial strain improvement processes.

## Discussion

Large-scale whole-genome sequencing and phylogenomic analysis is increasingly used for identifying targets for genome and metabolic engineering, studies of metabolic capabilities, pathogen phylogenomics and evolutionary studies. These studies are often complicated by the large number of sequences in the databases, database redundancy and the poor quality of some genome sequence data. The development of the high-quality, curated ActDES, reported here, enables phylum-wide taxonomic representation of the *Actinobacteria* coupled with quality-filtered genome data and equivalent annotation for each CDS.

The intended primary use of ActDES will be in the study of primary metabolism, but it is not limited. It can also inform the development and evolution of metabolism in strains that produce bioactive metabolites, given the high representation of genera renowned for their ability to produce natural products such as *Streptomyces* and *Micromonospora *. Due to a greater understanding of BGC evolution and genome organization in *Actinobacteria *, it is becoming increasingly clear that genes whose functions are in primary metabolism may actually contribute directly to the biosynthesis of specialized metabolites and, hence, the identification of duplicates may indicate the presence of cryptic BGCs [6, 11] or, when associated with precursor biosynthetic genes, provide the raw material for the enzymes across multiple BGCs [32–34].

ActDES may also find utility in evolutionary studies of expanded gene families across the actinobacterial phylum that contribute to virulence, such as the *mce* locus, which is known to facilitate host survival in mycobacteria [35], but also facilitates xenobiotic substrate uptake in *Rhodococcus* [36], and enables root colonization and survival in *Streptomyces* [37]. With phylum-wide taxonomic representation of established actinobacterial animal and plant pathogens, the scope for evolutionary studies using these data is enormous.

### Usage notes

The CVS files of each genome contain the rast annotation details in addition to the DNA and protein sequences for each annotated CDS (https://doi.org/10.6084/m9.figshare.12167880). The genome list contains the rast ID (which is equivalent to the name of the .cvs file) along with the NCBI ID (sequence ID; Table S1) plus the species name, which are included in the dataset. Further details of annotating batches of genomes in rast can be found at https://github.com/nselem/myrast.

The primary metabolism expansion tables (Table S2) are organized by metabolic pathway along the top row with the Enzyme Commission (EC) number and functional annotation, with the first column being the taxonomic assignment. The genus table shows the mean number of genes of the annotated function. Highlighted cells reflect gene expansion events, i.e. those genes that are present in a higher number than the overall mean across the database plus the standard deviation.

It is suggested that the gene expansion table (Table S2) is searched in the first instance (either by species or genus of interest or by a specific enzymatic function). This can be carried out by a simple text search. This will then allow the identification of a query sequence from a species or gene of interest (either nucleotide or amino acid sequence), which can then be searched against the curated blast database allowing a detailed phylogenetic analysis of a gene/protein of interest by using standard alignment and tree building software tools. These data can also be used in detailed evolutionary analysis of selection, mutation rates, etc. We have set up a Jupyter Notebook through the MyBinder project (https://mybinder.org/) to enable ease of use of the code (https://github.com/nselem/ActDES) with a tutorial to enable use of the database (https://github.com/nselem/ActDES).

## References

[1] Mukherjee S, Seshadri R, Varghese NJ, Eloe-Fadrosh EA, Meier-Kolthoff JP (2017). 1003 reference genomes of bacterial and archaeal isolates expand coverage of the tree of life. Nat Biotechnol.

[2] Wu D, Hugenholtz P, Mavromatis K, Pukall R, Dalin E (2009). A phylogeny-driven genomic encyclopaedia of Bacteria and Archaea. Nature.

[3] Kunin V, Cases I, Enright AJ, de Lorenzo V, Ouzounis CA (2003). Myriads of protein families, and still counting. Genome Biol.

[4] Goodfellow M (2015). Bergey’s Manual of Systematics of Archaea and Bacteria.

[5] Ventura M, Canchaya C, Tauch A, Chandra G, Fitzgerald GF (2007). Genomics of actinobacteria: tracing the evolutionary history of an ancient phylum. Microbiol Mol Biol Rev.

[6] Chevrette MG, Gutiérrez-García K, Selem-Mojica N, Aguilar-Martínez C, Yañez-Olvera A (2020). Evolutionary dynamics of natural product biosynthesis in bacteria. Nat Prod Rep.

[7] Adamek M, Alanjary M, Ziemert N (2019). Applied evolution: phylogeny-based approaches in natural products research. Nat Prod Rep.

[8] Ziemert N, Alanjary M, Weber T (2016). The evolution of genome mining in microbes – a review. Nat Prod Rep.

[9] Medema MH, Fischbach MA (2015). Computational approaches to natural product discovery. Nat Chem Biol.

[10] Adamek M, Alanjary M, Sales-Ortells H, Goodfellow M, Bull AT (2018). Comparative genomics reveals phylogenetic distribution patterns of secondary metabolites in *Amycolatopsis* species. BMC Genomics.

[11] Cruz-Morales P, Kopp JF, Martínez-Guerrero C, Yáñez-Guerra LA, Selem-Mojica N (2016). Phylogenomic analysis of natural products biosynthetic gene clusters allows discovery of arseno-organic metabolites in model streptomycetes. Genome Biol Evol.

[12] Navarro-Muñoz JC, Selem-Mojica N, Mullowney MW, Kautsar SA, Tryon JH (2020). A computational framework to explore large-scale biosynthetic diversity. Nat Chem Biol.

[13] Sélem-Mojica N, Aguilar C, Gutiérrez-García K, Martínez-Guerrero CE, Barona-Gómez F (2019). EvoMining reveals the origin and fate of natural product biosynthetic enzymes. Microb Genom.

[14] Doroghazi JR, Metcalf WW (2013). Comparative genomics of actinomycetes with a focus on natural product biosynthetic genes. BMC Genomics.

[15] Schniete JK, Cruz-Morales P, Selem-Mojica N, Fernández-Martínez LT, Hunter IS (2018). Expanding primary metabolism helps generate the metabolic robustness to facilitate antibiotic biosynthesis in *Streptomyces*. mBio.

[16] Edgar RC (2004). MUSCLE: a multiple sequence alignment method with reduced time and space complexity. BMC Bioinformatics.

[17] Howe K, Bateman A, Durbin R (2002). QuickTree: building huge neighbour-joining trees of protein sequences. Bioinformatics.

[18] Minh BQ, Schmidt HA, Chernomor O, Schrempf D, Woodhams MD (2020). IQ-TREE 2: new models and efficient methods for phylogenetic inference in the genomic era. Mol Biol Evol.

[19] Ronquist F, Teslenko M, van der Mark P, Ayres DL, Darling A (2012). MrBayes 3.2: efficient Bayesian phylogenetic inference and model choice across a large model space. Syst Biol.

[20] Tang X, Li J, Millán-Aguiñaga N, Zhang JJ, O’Neill EC (2015). Identification of thiotetronic acid antibiotic biosynthetic pathways by target-directed genome mining. ACS Chem Biol.

[21] Schmidt KL, Peterson ND, Kustusch RJ, Wissel MC, Graham B (2004). A predicted ABC transporter, FtsEX, is needed for cell division in *Escherichia coli*. J Bacteriol.

[22] Steffensky M, Mühlenweg A, Wang Z-X, Li S-M, Heide L (2000). Identification of the novobiocin biosynthetic gene cluster of *Streptomyces spheroides* NCIB 11891. Antimicrob Agents Chemother.

[23] Kling A, Lukat P, Almeida DV, Bauer A, Fontaine E (2015). Targeting DnaN for tuberculosis therapy using novel griselimycins. Science.

[24] Peterson RM, Huang T, Rudolf JD, Smanski MJ, Shen B (2014). Mechanisms of self-resistance in the platensimycin- and platencin-producing *Streptomyces platensis* MA7327 and MA7339 strains. Chem Biol.

[25] Case RJ, Boucher Y, Dahllöf I, Holmström C, Doolittle WF (2007). Use of 16S rRNA and rpoB genes as molecular markers for microbial ecology studies. Appl Environ Microb.

[26] Fernández-Martínez LT, Hoskisson PA (2019). Expanding, integrating, sensing and responding: the role of primary metabolism in specialised metabolite production. Curr Opin Microbiol.

[27] Gubbens J, Janus M, Florea BI, Overkleeft HS, van Wezel GP (2012). Identification of glucose kinase-dependent and -independent pathways for carbon control of primary metabolism, development and antibiotic production in *Streptomyces coelicolor* by quantitative proteomics. Mol Microbiol.

[28] van Wezel GP, König M, Mahr K, Nothaft H, Thomae AW (2007). A new piece of an old jigsaw: glucose kinase is activated posttranslationally in a glucose transport-dependent manner in *Streptomyces coelicolor* A3(2). J Mol Microb Biotech.

[29] Argimón S, Abudahab K, Goater RJE, Fedosejev A, Bhai J (2016). Microreact: visualizing and sharing data for genomic epidemiology and phylogeography. Microb Genom.

[30] Bertram R, Schlicht M, Mahr K, Nothaft H, Saier MH (2004). *In silico* and transcriptional analysis of carbohydrate uptake systems of *Streptomyces coelicolor* A3(2). J Bacteriol.

[31] Schniete JK, Reumerman R, Kerr L, Tucker NP, Hunter IS (2020). Differential transcription of expanded gene families in central carbon metabolism of *Streptomyces coelicolor* A3(2). Access Microbiol.

[32] Chan YA, Podevels AM, Kevany BM, Thomas MG (2009). Biosynthesis of polyketide synthase extender units. Nat Prod Rep.

[33] Pfeifer BA, Khosla C (2001). Biosynthesis of polyketides in heterologous hosts. Microbiol Mol Biol Rev..

[34] Zhang G, Li Y, Fang L, Pfeifer BA (2015). Tailoring pathway modularity in the biosynthesis of erythromycin analogs heterologously engineered in *E. coli*. Sci Adv.

[35] Arruda S, Bomfim G, Knights R, Huima-Byron T, Riley L (1993). Cloning of an *M. tuberculosis* DNA fragment associated with entry and survival inside cells. Science.

[36] Mohn WW, van der Geize R, Stewart GR, Okamoto S, Liu J (2008). The actinobacterial *mce4* locus encodes a steroid transporter. J Biol Chem.

[37] Clark LC, Seipke RF, Prieto P, Willemse J, van Wezel GP (2013). Mammalian cell entry genes in *Streptomyces* may provide clues to the evolution of bacterial virulence. Sci Rep.

[38] Aziz RK, Bartels D, Best AA, DeJongh M, Disz T (2008). The RAST server: Rapid Annotations using Subsystems Technology. BMC Genomics.

[39] Altschul SF, Gish W, Miller W, Myers EW, Lipman DJ (1990). Basic local alignment search tool. J Mol Biol.

